# Electroacupuncture improves recovery after hemorrhagic brain injury by inducing the expression of angiopoietin-1 and -2 in rats

**DOI:** 10.1186/1472-6882-14-127

**Published:** 2014-04-05

**Authors:** Hua-Jun Zhou, Tao Tang, Jian-Hua Zhong, Jie-Kun Luo, Han-Jin Cui, Qi-Mei Zhang, Jing-Hua Zhou, Qiang Zhang

**Affiliations:** 1 Institute of Neurology, China Three Gorges University, Yichang, Hubei, China; 2 Department of t Neurology, The First College of Clinical Medical Science, China Three Gorges University, Yichang, Hubei, China; 3 Department of the Intensive Care Unit, The First College of Clinical Medical Science, China Three Gorges University, Yichang, Hubei, China; 4 Institute of Integrative Medicine, Xiangya Hospital, Central South University, Changsha, Hunan, People’s Republic of China

**Keywords:** Electroacupuncture, Zusanli acupoint, Intracerebral hemorrhage, Angiogenesis, Angiopoietin

## Abstract

**Background:**

Angiopoietin (Ang) is one of the major effectors of angiogenesis, playing a critical role in neurovascular remodeling after stroke. Acupuncture has been widely used for treating stroke in China for a long time. Recently, we have demonstrated that electroacupuncture (EA) can accelerate intracerebral hemorrhage (ICH)-induced angiogenesis in rats. In the present study, we investigated the effect of EA on the expression of Ang-1 and Ang-2 in the brain after ICH.

**Methods:**

ICH was induced by stereotactic injection of collagenase type VII into the right globus pallidus. Adult male Sprague–Dawley rats were randomized into the following four groups: sham-operation (SHAM), stroke-no electroacupuncture (SNE), stroke-EA at the Zusanli acupoint (SEZ), and stroke-EA at a nonacupoint (SEN). EA was applied to the bilateral Zusanli (ST36) acupoint in the SEZ group and a nonacupoint in the SEN group. The expression of Ang-1 and Ang-2 was evaluated by immunohistochemistry and quantitative real-time reverse transcription-polymerase chain reaction (RT-PCR).

**Results:**

Some Ang-1 and Ang-2 immunoreactive microvessels with a dilated outline were detected in the perihematomal tissues after ICH, and the vessels extended into the clot from the surrounding area since day 7. The expression of Ang-1 increased notably as long as 2 weeks after ICH, while Ang-2 immunoreactivity declined at about 7 days following a striking upregulation at 3 days. EA at the Zusanli (ST36) acupoint upregulated the expression of Ang-1 and Ang-2 at both the protein and mRNA levels. However, EA at a nonacupoint had little effect on the expression of Ang-1 and Ang-2.

**Conclusions:**

Our data suggest that EA at the Zusanli (ST36) acupoint exerts neuroprotective effects on hemorrhagic stroke by upregulation of Ang-1 and Ang-2.

## Background

Intracerebral hemorrhage (ICH) is a lethal stroke type, as mortality approaches 50% and neurological disability in survivors is common [1]. Despite decades of intense research, current treatments for minimizing disability and mortality after ICH are far from satisfactory.

Angiogenesis, the outgrowth of new vessels from pre-existing vasculature, may play a critical role in neurovascular remodeling, which is a key component of recovery after stroke [2]. Furthermore, it is currently considered that angiogenesis promotes neurogenesis [3] and that regrowth of vascular structures might provide the requisite molecular (as well as anatomic) support for recovering neural networks [4].

The angiopoietin family, including Ang-1 and Ang-2, is one of the major effectors of angiogenesis. These molecules carry out signaling through the receptor tyrosine kinase Tie-2. Ang-1 is expressed on endothelial cells [5] and has been shown to promote the stabilization, maturation, and remodeling of vascular networks in the brain [6]. In contrast, Ang-2 is thought to destabilize vascular networks by inhibiting the Ang-1/Tie-2 interaction [7].

Electroacupuncture (EA) is a traditional therapy that has been widely applied for the treatment of hemorrhagic stroke because it has been shown to improve the outcome in experimental animals [8,9] and clinical practice [10]. Our previous studies have demonstrated that angiogenesis can occur in rat brains with ICH [11-13]. Moreover, we have recently demonstrated that EA can accelerate ICH-induced angiogenesis in rats and upregulate the expression of hypoxia-inducible factor-1α [14]. However, to date, there have been few reports regarding the effect of EA on the expression of Ang-1 and Ang-2 after ICH. In the present study, we investigated whether EA at the Zusanli (ST36) acupoint improves recovery after ICH by enhancing the expression of Ang-1 and Ang-2 in rats, thereby elucidating the mechanism of EA to enhance ICH-induced angiogenesis and providing new evidence for the application of acupuncture for the treatment of ICH.

## Methods

### Animal preparation

Studies were carried out on adult male Sprague–Dawley rats (250–300 g, 8–10 weeks of age) obtained from the Experimental Animal Science Center of Central South University, which were housed under identical conditions (room temperature at 25°C, 12 h light–dark cycle) and allowed free access to food and water. The experiment was performed in compliance with the guidelines of Central South University and the National Institute of Health Guide for the Care and Use of Laboratory Animals (NIH Publication No. 80–23); and the protocol was approved by the Institutional Animal Care and Use Committee of Central South University (2008-xy-0116). Rats with collagenase-induced ICH were randomly divided into four groups (n = 30 in each group): sham-operation group (SHAM), stroke-no EA (SNE), stroke-EA at the Zusanli acupoint (SEZ), and stroke-EA at a nonacupoint (SEN).

### Induction of intracerebral hemorrhage

ICH was induced with collagenase according to a previous protocol [15]. After anesthetization with chloral hydrate (400 mg/kg) via intraperitoneal injection, the animals were fixed in the prone position on a stereotactic frame (Stoelting Co., USA). Following a scalp incision, a small cranial burr was drilled near the right coronal suture at 3.2 mm lateral to the midline. Bacterial type VII collagenase (0.5 U in 2.5 μL of 0.9% sterile saline, Sigma Co., USA) was slowly injected into the right globus pallidus (1.4 mm posterior and 3.2 mm lateral to the bregma, 5.6 mm ventral to the cortical surface) with a 5-μL Hamilton syringe for at least 5 min, and the needle remained there for another 5 min. The bone hole was sealed with bone wax, and then the wound was sutured. The animals were placed in a warm box to recover individually. For the SHAM group, the rats were injected with 2.5 μL of 0.9% sterile saline without collagenase at the same site. During the procedure, the rectum temperature was monitored and maintained at 37.5°C with a feedback-controlled heating pad.

### Electro-acupuncture treatment

The rats were placed into tailor-made mouse cages, and their bilateral legs were sufficiently exposed. Stainless acupuncture needles of 0.3 mm in diameter were bilaterally inserted at a depth of about 2–4 mm into the locus of the Zusanli (ST36) acupoint, which was located at 5 mm lateral and distal to the anterior tubercle of the tibia in the SEZ group. The needles were inserted into the hip in the SEN group. The rats were acupunctured with an electrical needle stimulator (WQ1002K, Electro-Acupuncture Equipment Company, China) at 9–10 am, 30 min each time for 14 days. The frequency was 2–20 Hz. The original intensity was 2 V and was increased by 1 V every 10 min. All rats were conscious when EA was performed. No stimulation was given to the SNE and SHAM groups.

### Neurological evaluation

According to the study reported by Hua et al. [16], the use of a forelimb asymmetry test was adopted. Every rat was laid in a transparent cylinder, 20 cm in diameter and 30 cm in height. A mirror was placed near the cylinder at an angle convenient for observing the forelimb movement of the rat; and at the same time, simultaneous recording was performed by a video camera. The test lasted 10 min. In the testing time, the rat behavior was quantified by counting the occasions that the rat's forelimb touched the cylinder wall while it was in an orthostatic position with a balanced gravity center: the occasions of contact with the unimpaired (ipsilateral) forelimb were recorded as I, that with the impaired forelimb (contralateral to the collagenase injection site) as C, and with both forelimbs as B. Then, the forelimb asymmetric use rate (AUR) was calculated by the following formula: AUR = [I/(I + C + B)] - [C/(I + C + B)].

### Immunohistochemistry

Under deep anesthesia with chloral hydrate (800 mg/kg), the collagenase-induced ICH animals (n = 5, per time point) were randomly chosen at day 3, day 7, and day 14 postoperation, and they were transcardially perfused with 0.9% saline followed by 250 mL of ice-cold 4% paraformaldehyde in 0.1 M phosphate buffer (pH = 7.4). The brains were removed and post-fixed in the same fixative for 2 h, and then sequentially transferred to 20% and 30% sucrose in 0.1 M phosphate buffer (pH = 7.4) at 4°C until sinking. The brains were cut for 30-μm coronal sections at -20°C with a cryostat (CM1900, Leica Co., Germany) for immunohistochemical staining.

Sections were brought to room temperature and incubated in 3% H_2_O_2_ in methanol for 15 min. After washing three times in phosphate-buffered saline for 5 min each, nonspecific binding was blocked in 5% bovine serum albumin (BSA, Sigma, USA) for 1 h at 37°C. Sections were not washed, incubated with goat anti-Ang-1 (Santa Cruz Biotech, 1:100) or goat anti-Ang-2 (Santa Cruz Biotech, 1:100) overnight at 4°C, then with a biotinylated anti-goat IgG (1:100) for 1 h. Color development was performed with a Vectastain ABC kit (Vector Laboratories), according to the vendor’s protocol. As a negative control, 1% BSA was used instead of the primary antibody. The mean optical densities (MODs) of HIF-1α-, VEGF-, Ang-1-, and Ang-2-positive microvessels were measured quantitatively within the striatum adjacent to the hematoma with the Motic Images Advance 3.2 image analysis system.

### Quantitative real-time reverse transcription-polymerase chain reaction (RT-PCR)

Total RNA was purified from 100 mg of tissue near the hematoma in each group using the TRIZOL Reagent (Invitrogen, Carlsbad, CA). The integrity of the total RNA was detected by agarose gel electrophoresis; the purity and concentration were detected by a spectrophotometer (UV-1201, Shimadzu). Reverse transcription was performed with 2 μg of total RNA using 1 μg/μL oligo(dT)18 (1 μL), 10 mM dNTP Mix (2 μL), RNase inhibitor (1 μL), and 200 U/μL M-Mulv-Reverse Transcriptase (1 μL) at 70°C for 5 min, 37°C for 5 min, 42°C for 60 min, and 70°C for 10 min, following the manufacturer’s instructions (Fermentas, CA). The cDNA was stored at -20°C. PCR amplification was performed using a SYBR Premix ExTaq™ PCR kit (4 μL of 1:2 cDNA dilution was used, Takara Biotechnology, Japan) in a LightCycler Real-Time Detection System (Roche Diagnostics Limited, Germany). The following thermocycling protocol was used: 10 s at 95°C; 30–40 cycles of 5 s at 95°C, 20 s at 52°C, and 10 s at 72°C; and a melting curve at 60°C. Primers for Ang-1, Ang-2, and β-actin were designed with Primer Premier 5.0 software for the rat (PRIMER Biosoft International, CA) as follows: Ang-1, sense 5′- CACCGTGAGGATGGAAGCCTA-3′ and antisense 5′- TTCCCAAGCCAATATTCACCAGA-3′; Ang-2, sense 5′- CAGTAGCATCAGCCAACCAGGA-3′ and antisense 5′- GACCACATGCGTCGAACCAC-3′; β-actin, sense 5′- CGTTGACATCCGTAAAGAC-3′ and antisense 5′- TGGAAGGTGGACAGTGAG-3′. Melting curves of all samples were performed as controls of specificity. All gene expression data were calculated as 2^-ΔΔCT^ (n = 5), which indicates an n-fold change in gene expression relative to the SHAM control sample [17].

### Statistical analysis

All data in this study are presented as mean ± standard deviation (SD). Data were analyzed by the Student’s *t*-test and one-way analysis of variance, followed by Scheffe’s *post-hoc* test. Differences were considered significant at *P <* 0.05.

## Results

### Neurological evaluation

**Figure 1:**
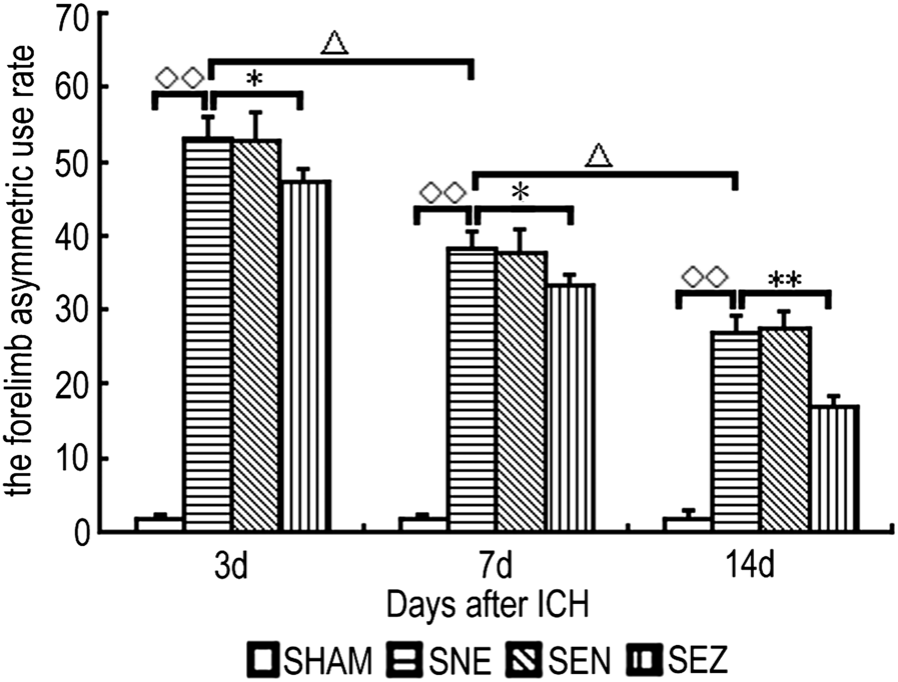
**Neurological evaluation scores.** The forelimb asymmetric use rate (AUR) of the SNE group gradually decreased as the observation time progressed, and all AUR values were greater than those in the SHAM group at the corresponding time points. In addition, the AUR values of the SEZ group were significantly less than those of the SNE group. However, EA at a nonacupoint had little effect on the neurological recovery after ICH. ^◇◇^*P <* 0.01 vs. the SHAM group; *P <* 0.05, **P < 0.01 vs. the SNE group. ^△^*P <* 0.05 vs. the former time point in the SNE group (mean ± SD, n = 5 for each time point).

After ICH, the rats were observed for neurological deficits: the AUR gradually decreased as the observation time progressed (*P* < 0.05), and the AUR values of the SNE, SEN, and SEZ groups were greater than those in the SHAM group at the corresponding time points (*P* < 0.01). A subsequent analysis revealed that the AUR values of the SEZ group were significantly less than those of the SNE group (*P* < 0.05). However, EA at a nonacupoint had little effect on the neurological recovery after ICH (Figure 1).

### Overexpression of Ang-1 and Ang-2 after EA

**Figure 2:**
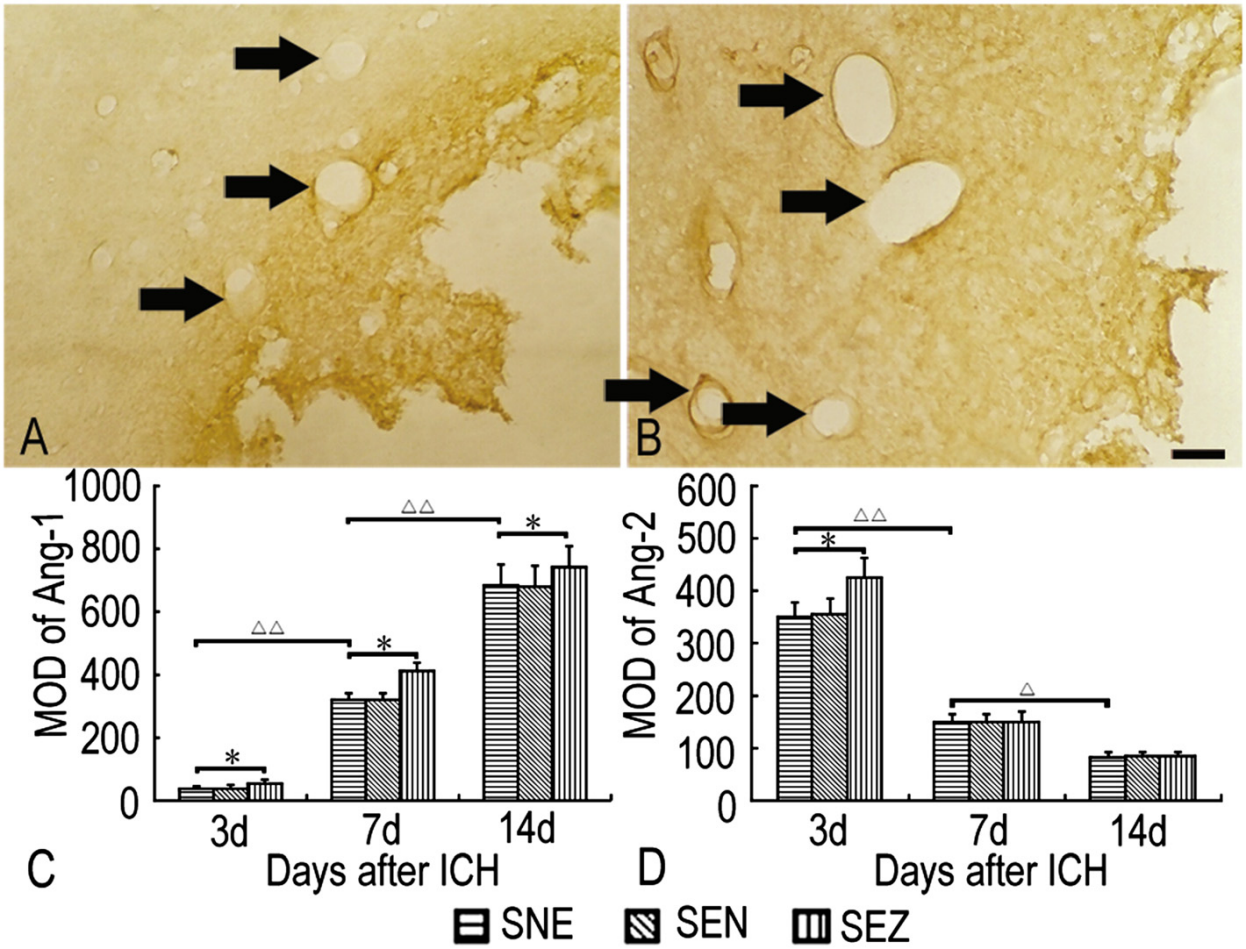
**Immunohistochemistry for detection of Ang-1 and Ang-2 after ICH.** After ICH, some Ang-1- **(A)** and Ang-2- **(B)** positive microvessels of the enlarged profile were detected in the perihematomal tissue after ICH. Ang-1 immunoreactivity increased notably as long as 2 weeks after ICH **(C)**, while Ang-2 immunoreactivity declined at about 7 days following a striking upregulation at 3 days **(D)**. EA at the Zusanli (ST36) acupoint increased Ang-1 and Ang-2 expression as compared with no EA treatment after ICH. Meanwhile, there was no significant difference in the expression of Ang-1 and Ang-2 between the SEN group and the SNE group. Scale bar = 100 μm. *P < 0.05 vs. the SNE group. ^△^*P <* 0.05, ^△△^*P <* 0.01 vs. the former time point in the SNE group (mean ± SD, n = 5 for each time point).

At 3 days after ICH, some Ang-1- and Ang-2-positive dilated vessels were detected mainly in the perihematomal tissue, and these positive vessels extended into the clot from 7 days (Figure 2A and B). However, only Ang-1-positive vessels were occasionally observed in sham-operated animals.

Ang-1 immunoreactivity increased notably as long as 2 weeks after ICH (*P <* 0.01), while Ang-2 immunoreactivity declined at about 7 days following a striking upregulation at 3 days (*P <* 0.05). EA at the Zusanli (ST36) acupoint increased Ang-1 expression notably from 3 days to 14 days and Ang-2 expression only at 3 days as compared with no EA treatment after ICH (*P < 0.05*). Meanwhile, there was no significant difference in the expression of Ang-1 and Ang-2 between the SEN group and the SNE group (Figure 2C and D).

### Increased mRNA Expression of Ang-1 and Ang-2 after EA

**Figure 3:**
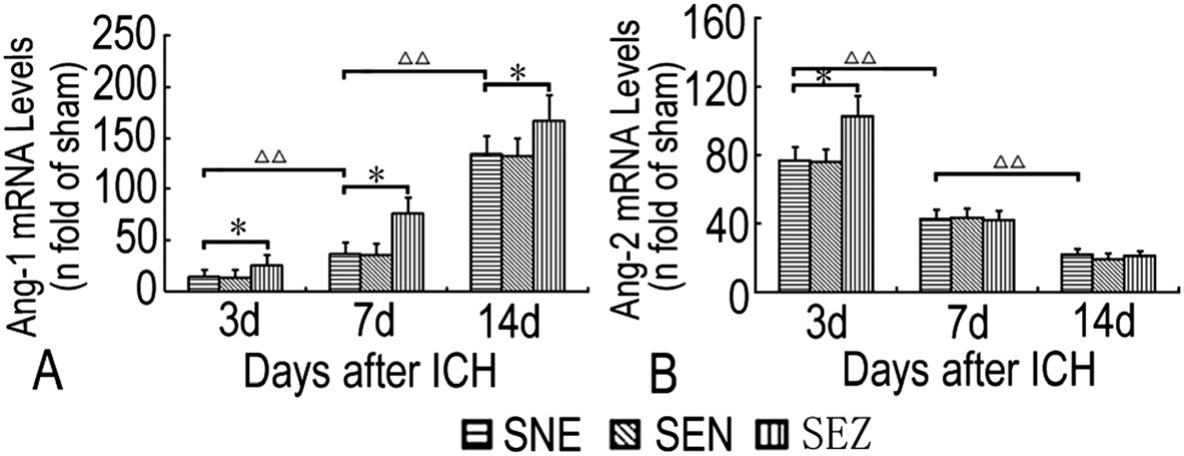
**Quantitative analysis of Ang-1 and Ang-2 mRNA after ICH.** After ICH induction, notable upregulation of Ang-1 **(A)** and Ang-2 **(B)** mRNAs could be detected at 3 days, and the upregulation of Ang-1 persisted until 14 days, while the expression of Ang-2 mRNA decreased after 7 days. *Post-hoc* analysis indicated striking increases in the expression of Ang-1 mRNA from 3 days to 14 days and Ang-2 mRNA at 3 days in the SEZ group as compared with the SNE group. However, EA at a nonacupoint had little effect on the expression of Ang-1 and Ang-2 at the mRNA level. *P < 0.05 vs. the SNE group.^△△^*P <* 0.01 vs. the former time point in the SNE group (mean ± SD, n = 5 for each time point).

Weak Ang-1 mRNA signals and no Ang-2 mRNA signals were detected in sham-operated rats. However, after ICH induction, notable upregulation of Ang-1 (Figure 3A) and Ang-2 (Figure 3B) mRNA could be detected at 3 days, and the upregulation of Ang-1 mRNA persisted until 14 days (*P <* 0.01), while the expression of Ang-2 mRNA decreased after 7 days (*P <* 0.01). *Post-hoc* analysis indicated striking increases in expression of Ang-1 mRNA from 3 days to 14 days and Ang-2 mRNA at 3 days in the SEZ group as compared with the SNE group (*P < 0.05*). However, EA at a nonacupoint had little effect on the expression of Ang-1 and Ang-2 at the mRNA level (Figure 3).

## Discussion

The present study demonstrated that EA can exert neuroprotective effects directly against hemorrhagic injury in rats. ICH upregulated the expression of Ang-1 and Ang-2, and EA further increased their expression.

It is well known that angiogenic factors play an essential role in regulating angiogenesis. Previous studies have demonstrated that angiogenesis in the brain occurs normally during early development [18] and can occur under pathological conditions such as ischemia [19,20] or hemorrhage [11-13]. Along with the development of neurovascularization, the role of angiogenesis in the recovery of stroke has received increasing attention in recent years. Ang-1 and Ang-2 have been shown to take part in regulating ischemia-induced angiogenesis in rat brains [19,20].

Numerous studies have demonstrated that acupuncture at the Zusanli (ST36) acupoint possesses a neuroprotective effect by suppressing neuron apoptosis [21,22], improving neural plasticity [23], increasing cerebral blood flow, and improving microcirculation [24] in ischemic rat brains. Recently, acupuncture at the Zusanli acupoint has been shown to not only suppress ICH-induced apoptotic neuronal cell death [8] but also accelerate ICH-induced angiogenesis in rats [14].

The present study showed a remarkable increase in the expression of Ang-1 and Ang-2 after ICH. These changes come not only from the induction of ICH, but, more significantly, from EA at the Zusanli acupoint. A robust upregulation of Ang-2 at the earliest stages of ICH caused by EA may destabilize the vessel and promote disassembly of the cellular components, allowing the formation of new vessels to occur in the endothelium [25,26]. In addition, the upregulation of Ang-1 resulting from EA might reflect an initial attempt to protect the peripheral vasculature from leakage at the early stage [27] and promote the stabilization and maturation of new vessels at the late stage [6].

It is common knowledge that ICH can cause increases of many inflammatory factors such as tumor necrosis factor-α and interleukin [28] as well as induce excessive nitric oxide (NO) production [29]. Several studies have shown that NO can upregulate Ang-1 expression after stroke, and the effects of inflammatory cytokines on the expression of Ang-1 and Ang-2 depend on their concentration: high concentrations of cytokines result in negative expression of Ang-1 and Ang-2, while low concentrations of cytokines result in positive expression of Ang-1 and Ang-2 [30-33]. Furthermore, EA can upregulate the NO level [34] and have an anti-inflammatory effect after stroke [35]. Accordingly, it is tempting to suppose that EA at the Zusanli (ST36) acupoint regulates the expression of Ang-1 and Ang-2 by increasing the NO level and decreasing inflammation. ICH also leads to upregulation of endothelial nitric oxide synthase (eNOS) [36], which is responsible for the angiogenic action of Ang-1 [37]. Moreover, EA can upregulate the eNOS level [38]. Hence, the upregulation of Ang-1 induced by EA after ICH may exert its angiogenic action through eNOS.

Angiogenesis is a complex process that requires orchestrated effects of many growth factors. Other than the Ang family, there are many pro-angiogenic (e.g., fibroblast growth factor, transforming growth factor) and anti-angiogenic (e.g., endostatin, thrombospondin) growth factors [39]. Thus, EA-induced neuroprotection in hemorrhagic stroke probably also involves these mechanisms. However, further studies are needed to provide direct evidence showing the causal relationship between EA-induced motor improvement and cellular expression of angiogenesis. In addition, understanding how this effect is regulated *in vivo* by the interaction with other growth factors is a continuing challenge.

## Conclusions

In summary, EA in rats subjected to ICH improves neurological recovery, which may be associated with angiogenesis and the expression of angiogenic factors. Moreover, the upregulation of Ang-1 and Ang-2 triggered by EA may act as one of the mechanisms of EA-induced neuroprotection in stroke.

## Abbreviations

Ang: Angiopoietin; EA: Electroacupuncture; eNOS: Endothelial nitric oxide synthase; ICH: Intracerebral hemorrhage; NO: Nitric oxide.

## Competing interests

The authors declared that they have no competing interests.

## Authors’ contributions

Author contributions to the study and manuscript preparation are as follows. Conception and design: TT and HJZ. Carried out the experiments: HJZ, JHZ, and HJC. Acquisition of data: JKL, QMZ, JHZ, and QZ Analysis and interpretation of data: TT and HJZ. Drafting the article: HJZ. Critically revised the article: all authors. Reviewed submitted version of the manuscript: all authors. Approved the final version of the manuscript on behalf of all authors: TT Statistical analysis: HJZ Study supervision: TT.

## Pre-publication history

The pre-publication history for this paper can be accessed here:

http://www.biomedcentral.com/1472-6882/14/127/prepub
